# Effect of feeding Chinese herb medicine ageratum-liquid on intestinal bacterial translocations induced by H9N2 AIV in mice

**DOI:** 10.1186/s12985-019-1131-y

**Published:** 2019-02-21

**Authors:** Haoran Lu, Luxuan Zhang, Junfang Xiao, Che Wu, Huanmin Zhang, Yihu Chen, Zhengyong Hu, Wencheng Lin, Qingmei Xie, Hongxin Li

**Affiliations:** 1 Guangdong experimental high school, Guangzhou, 510145 Gaungdong China; 2 College of Animal Science, South China Agricultural University and Guangdong Provincial Key Laboratory of Agro-Animal Genomics and Molecular Breeding and Key Laboratory of Chicken Genetics, Breeding and Reproduction, Ministry of Agriculture, Guangzhou, 510642 People’s Republic of China; 3 Key Laboratory of Animal Health Aquaculture and Environmental Control, Guangdong Guangzhou, 510642 People’s Republic of China; 4 USDA, Agriculture Research Service, Avian Disease and Oncology Laboratory, East Lansing, MI 48823 USA

**Keywords:** H9N2 avian influenzas virus, Intestinal microbiota, Translocation, Chinese herb medicine, Ageratum-liquid

## Abstract

**Background:**

As a low pathogenic influenza virus, avian influenza virus subtype H9N2 (H9N2 AIV) often induces high morbidity in association with secondary bacterial infections in chickens or mammals. To explore this phenomenon, the relationship between intestinal microflora changes and bacterial translocations was studied post H9N2 AIV challenge and post AIV infection plus Ageratum-liquid treatment.

**Methods:**

Illumina sequencing, histological examination and Neongreen-tagged bacteria were used in this study to research the microbiota composition, intestinal barrier, and bacterial translocation in six weeks of BALB/c mice.

**Results:**

H9N2 AIV infection caused intestinal dysbacteriosis and mucosal barrier damages. Notably, the villus length was significantly reduced (*p* < 0.01) at 12 dpi and the crypt depth was significantly increased (*p* < 0.01) at 5 dpi and 12 dpi with infection, resulting in the mucosal regular villus-length/crypt-depth (V/C) was significantly reduced (*p* < 0.01) at 5 dpi and 12 dpi. Moreover, degeneration and dissolution of the mucosal epithelial cells, loose of the connective tissue and partial glandular atrophy were found in infection group, indicating that intestinal barrier function was weakened. Eventually, intestinal microbiota (*Staphylococcus*, *E. coli*, etc.) overrun the intestinal barrier and migrated to liver and lung tissues of the mice at 5 and 12 dpi. Furthermore, the bacteria transferred in mesentery tissue sites from intestine at 36 h through tracking the Neongreen-tagged bacteria. Then the Neongreen-tagged bacteria were isolated from liver at 48 h post intragastrical administration. Simultaneously, Ageratum-liquid could inhibit the intestinal microbiota disorder post H9N2 AIV challenge via the respiratory tract. In addition, this study also illustrated that Ageratum-liquid could effectively prevent intestinal bacterial translocation post H9N2 AIV infection in mice.

**Conclusion:**

In this study, we report the discovery that H9N2 AIV infection could damage the ileal mucosal barrier and induce the disturbance of the intestinal flora in BALB/c mice resulting in translocation of intestinal bacteria. In addition, this study indicated that Ageratum-liquid can effectively prevent bacterial translocation following H9N2 infection. These findings are of important theoretical and practical significance in prevention and control of H9N2 AIV infection.

**Electronic supplementary material:**

The online version of this article (10.1186/s12985-019-1131-y) contains supplementary material, which is available to authorized users.

## Background

Avian influenza virus subtype H9N2 (H9N2 AIV) is usually found in chicken, but also isolated from the mammals, even humans [1, 2]. Importantly, most high pathogenic AIV (e.g. H7N9) acquired their internal’ gene segments from H9N2 strains [3]. And the virus can be isolated from host tissues including trachea, lung, brain, spleen, pancreas, cloacal cavity and intestinal tract. However, no characteristic clinical symptoms and lesions were observed in animals individually infected with H9N2 AIV. Generally, hosts begin to develop inflammation and enteric problems at 3–5 days post infection, often resulting in morbidity or mortality due to infection with secondary bacteria, for example *Escherichia coli* [4]. In mammals, AIV H9N2 always causes mild respiratory illness, but fatal outcomes are sometimes observed [3, 5, 6]. After H9N2 AIV infection, the inflammation was serious, even with severe peritonitis, perihepatitis and pericarditis in chicken. The problem of bacterial secondary infection has also plagued many scholars and clinical practitioners, and also brought great difficulties to the prevention and control of the bird flu. This is a puzzle that animal infection with influenza virus would appear serious bacterial secondary infection and how do these bacteria break through the body’s mucosal barrier into vital organs? All of those questions deserve further research.

The intestinal tract is the body’s largest immune organ. Once the mucosal barrier and microbial flora are destroyed, the intestinal diseases will occur. The avian influenza virus can be replicated in intestinal epithelial cells after infection and will trigger the massive expression of inflammatory factors and appear intestinal inflammatory injury [7, 8]. In clinically, antibiotics were always used to treat. Antibiotics can kill bacteria, but can also cause damage to intestinal beneficial flora, aggravating intestinal injury because of its broad spectrum. A large number of studies have showed that Chinese herbal medicine in the regulation of the intestinal tract have a good effect, such as Ageratum-liquid (AL), which can regulate gastrointestinal motility function, repair damaged mucosa and reduce intestinal permeability [9, 10]. As a representative natural medicine, AL is considered safe and with few adverse drug reactions. Clinical studies found that AL has significant effect on the treatment of gastrointestinal influenza [11, 12]. In the view of these problems, this project, on the basis of previous studies, we used BALB/c mice as an animal model to infect H9N2 AIV with low pathogenicity to investigate the mechanism of H9N2 AIV promoting bacterial translocation in the intestine of mice infected with H9N2 AIV, the close relationship between intestinal bacterial translocation and secondary bacterial infection, and the feasibility of compound traditional Chinese medicine in preventing intestinal flora translocation to prevent secondary infection.

## Materials and methods

### Viral and bacterial strains

The H9N2 AIV strain A/mink/China/01/2014 (H9N2) was isolated from minks and obtained from Professor Qingfang Liu, Chinese Acad Agr Sci, Shanghai Vet Res Inst, Shanghai, Peoples R China. The viral titer was 10^6.1^ EID_50_/0.1 mL. Neongreen specific marker *Escherichia coli* (Neongreen-tagged bacteria) was obtained from Professor Youming Zhang, the State Key Laboratory of Microbial Technology, Shandong University, and the marker was visualized by a secreted Neongreen fluorescent reporter. Ageratum-Liquid (alcohol free) was used as a commercialized manufacture of Chinese herb medicine purchased from Taiji Group Chongqing Fuling Pharmaceutical Co., Ltd. (LOT number: 17021045, Approval Code: Z50020409, Chongqing, China) and used for treatment of the infection according the manufacturer’s instructions. Ageratum-Liquid is a Chinese patent medicine composed of 10 kinds of Chinese medical medicines, including Atractylodis Rhizoma, Citri Reticulatae Pericarpium, Magnoliae Ofcinalis Cortex, and Pinelliae Rhizoma, Angelicae Dahuricae Radix, Poria, and Arecae Pericarpium, Licorice extract, Patchouli oil and volatile oil in Perillae Folium, and the herbal prescription and reference compounds were relatively constant as previous description [10].

### Animals and experimental protocol

The six weeks of BALB/c mice with similar weight were housed in negative pressure isolator and supplied commercial food and water Ad libitum in an air-conditioned environment (22–24 °C) with a regular 12-h light/dark cycle. After 1 week of acclimatization, eighty mice were randomly divided into eight groups (Table 1). The animal well-being was monitored during whole experiment, and cloacal swabs of the mice were collected for virus isolation and detection after 3 days post H9N2 AIV infection. Five mice from Control, Infection, Ageratum-liquid and Infection-Ageratum-liqui groups were euthanized by CO_2_ asphyxiation recommended by previous description [13] at 5 and 12 dpi (day post infection) respectively. Then the distal ileal contents were collected in a sterile environment and frozen immediately in liquid nitrogen for 16S rRNA sequencing and four mice were randomly selected for posterior bacterial isolation in liver and lung. The other distal ileal (1 cm) was placed in the slice fixative to prepare pathological sections. In addition, three mice from Neongreen, Infected-Neongreen, Ageratum-liquid-Neongreen and Infection-Ageratum-liquid-Neongreen groups were euthanized as previous description at 12, 24, 36 and 48 h post the intragastrical administration and aseptic collection of liver, lung, mesentery and intestinal cavity contents in placed in sterile centrifuge tubes for posterior Neongreen-tagged isolation. All procedures involving the handling of animals were carried out in accordance with the Guide for the Care and Use of Laboratory Animals of the National Institutes of Health. The animal study protocol was approved by the South China Agricultural University Committee of Animal Experiments (approval ID: SYXK-2014-0136).

**Table 1:** Group of Experiments

Group	Treatment^1^
1 (Control)	PBS nose drops, 300 μL/per mouse
2 (Infection)	H9N2 AIV nose drops, 300 μL/per mouse
3 (Neongreen)	PBS nose drops, 300 μL/per mouse; intragastrical administration oflabeled bacteriaat 3 dpi, 300 μL/per mouse
4 (Infected-Neongreen)	H9N2 AIV nose drops, 300 μL/per mouse; intragastrical administration oflabeled bacteriaat 3 dpi, 300 μL/per mouse
5 (Ageratum-liquid)	PBS nose drops, 300 μL/per mouse; filled with ALat 2, 3, 4 dpi, 1 mL/100 g, perday
6 (Infection-Ageratum-liquid)	H9N2 AIV nose drops, 300 μL/per mouse; filled with ALat 2, 3, 4 dpi, 1 mL/100 g, perday
7 (Ageratum-liquid-Neongreen)	PBS nose drops, 300 μL/per mouse; filled with ALat 2, 3, 4 dpi, 1 mL/100 g, perday; intragastrical administration oflabeled bacteria at 3 dpi, 300 μL/per mouse
8 (Infection-Ageratum-liquid-Neongreen)	H9N2 AIV nose drops, 300 μL/per mouse; filled with ALat 2, 3, 4 dpi, 1 mL/100 g, perday; intragastrical administration oflabeled bacteria at 3 dpi, 300 μL/per mouse

Note:

^1^The titer of the virus was 106.1EID50/0.1 mL.Mice were anesthetized with a mixture of ketamine and xylazine (100 mg/10 mg/kg), suspended by an incisor wire on an angled stand, and then allowed to perform correlative treatment

### Virus isolation and identification

The total RNA was extracted by swab eluate and detected by RT-PCR. The PCR system was 20 μL volume, including10 μL of 2 × One-Step Buffer, 0.5 μL of primers pares (F: CAAGATGGAAGTAGTATCACT, R: TTGCCAATTATATACAAATGT), 2 μL of RNA, 1 μL of RNA, One-Step Enzyme Mix and 6 μL of ddH_2_O. Reaction procedure was followed: reverse transcription 30 min at 50 °C; Denaturation 5 min at 94 °C, 30 cycles, 10 s at 94 °C, 55 °C annealing, 15 s; 72 °C extend 90 s, it extends 10 min after 72 °C, preserve at 4 C. The amplified products were detected by 1% agarose gel electrophoresis, and the results were recorded by the VL gel imaging system (France). In addition, 20 mg ileal contents and lung were homogenized in 100 μL of phosphate buffered solution from each of Infection, Ageratum-liquid and Infection-Ageratum-liquid groups at 5 dpi and 12 dpi, and TCID_50_ on MDCK cells was used to determine virus titers.

### Bacterial isolation

Tissue samples including collected liver and lung of four mice were homogenized in 500 μL of sterile saline from each of Trial 1 and Trial 3 groups. 100 μL of grinding fluid was coated on the LB plate and cultured 48 h at 37 °C. The growth status of bacteria was observed.

### Neongreen-tagged bacteria isolation

200 mg tissue samples including collected liver, lung, mesentery and intestinal cavity contents of three mice were homogenized in 500 μL of sterile saline from each of 3, 4, 7 and 8 groups. 100 μL of grinding fluid was coated on the LA plate (containing ampicillin was 100 μg/ml) and cultured 48 h at 37 °C. The growth status of Neongreen-tagged bacteria was observed.

### Histological examination of intestinal segments and villus conditions

Intestinal sections were performed in a commercial company (Wuhan). The collected distal ileum was sectioned for evaluation of the ileal epithelium lesions as previous description [7]. Intestinal tissue slice was observed through the microscope (20 × 10). Ten complete structures of the villi and crypt depth were measured using Image-Pro Plus 6.0 (Media Cybernetics, Inc.), and V/C ratio were calculated.

### DNA extraction and 16S rRNA gene sequencing

Intestinal bacterial genomic DNA was extracted with a TIANamp Stool DNA Kit (TIANGEN, Beijing, China). Total DNA Trizol Reagent was used to extract DNA from all samples of the intestinal contents, according the manufacturer’s instructions. The DNA purity and concentration were determined using a NanoDrop spectrophotometer (Thermo, USA). A 16S rRNA were sequenced for the V4 region of 16S rRNA in BGI Science and Technology Service Co., Ltd. according to the previous description [7, 14].

### Data analyses

All the data were calculated by Excel 2016 and one-way ANOVA was performed by using SPSS 22.0 software. Multiple significant comparisons were analyzed by DUNCAN, *p* < 0.05 was considered as significant difference, the test data in average and standard deviation form.

## Results

### H9N2 AIV infection damaged the intestinal structure and induced intestinal bacterial translocations in mice

**Fig. 1:**
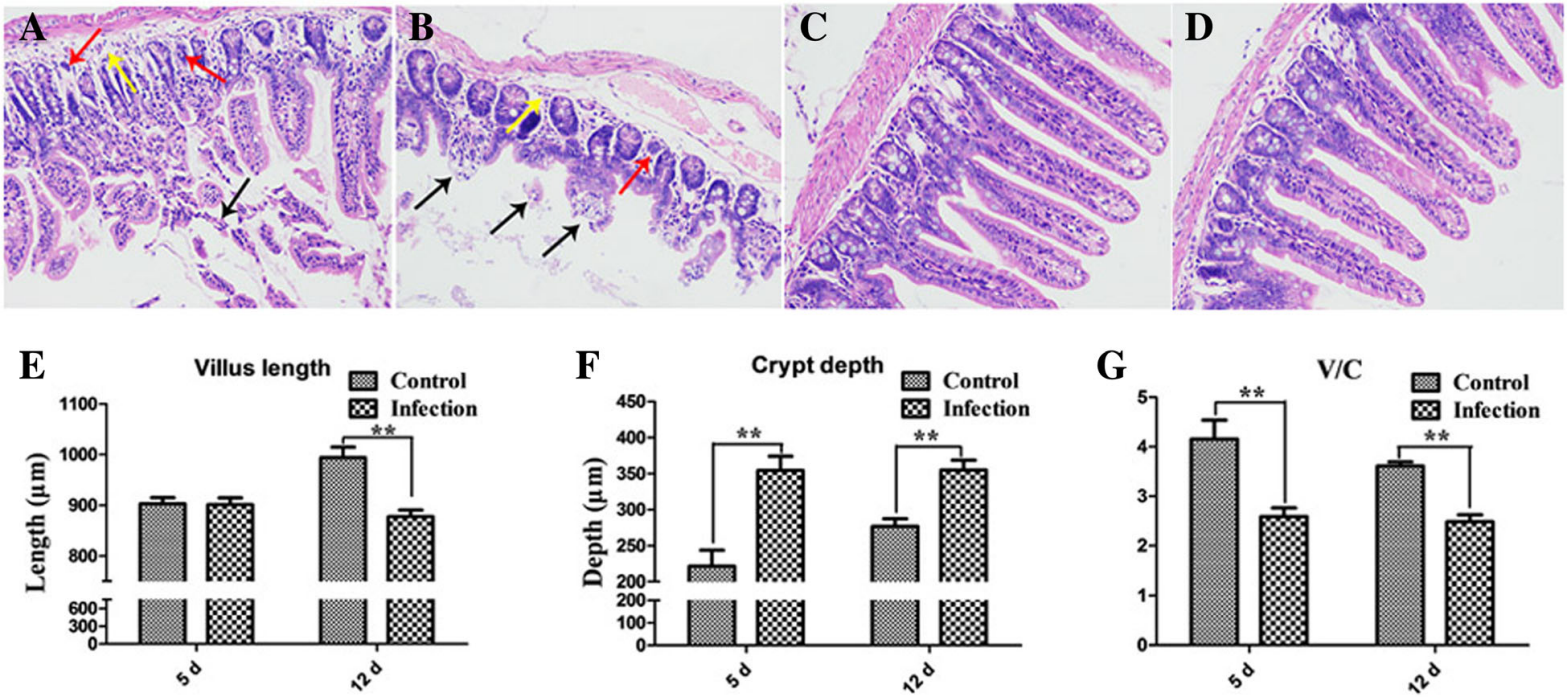
Histopathological changes in the ileal mucosa. **a**, **b** Histological features in the infection groups are shown with hematoxylin and eosin staining at 5 dpi and 12 dpi. Degeneration, dissolution and necrosis of the mucosal epithelial cells are indicated with the black arrow. Loose of the connective tissue is indicated with the yellow arrow. Pyknosis and atrophy of the intestinal glands are indicated with the red arrow. **c**, **d** Histological features in the control groups are shown with hematoxylin and eosin staining at 5 dpi and 12 dpi; **e** Measure of villus length by Image pro-plus, version 6.0 (Control = 5, Infection = 5). **f** Measure of crypt depth by Image pro-plus, version 6.0 (Control = 5, Infection = 5). **g** Spatial distribution of villus-length/crypt-depth of Control and Infection groups. ** *p* < 0.01

**Fig. 2:**
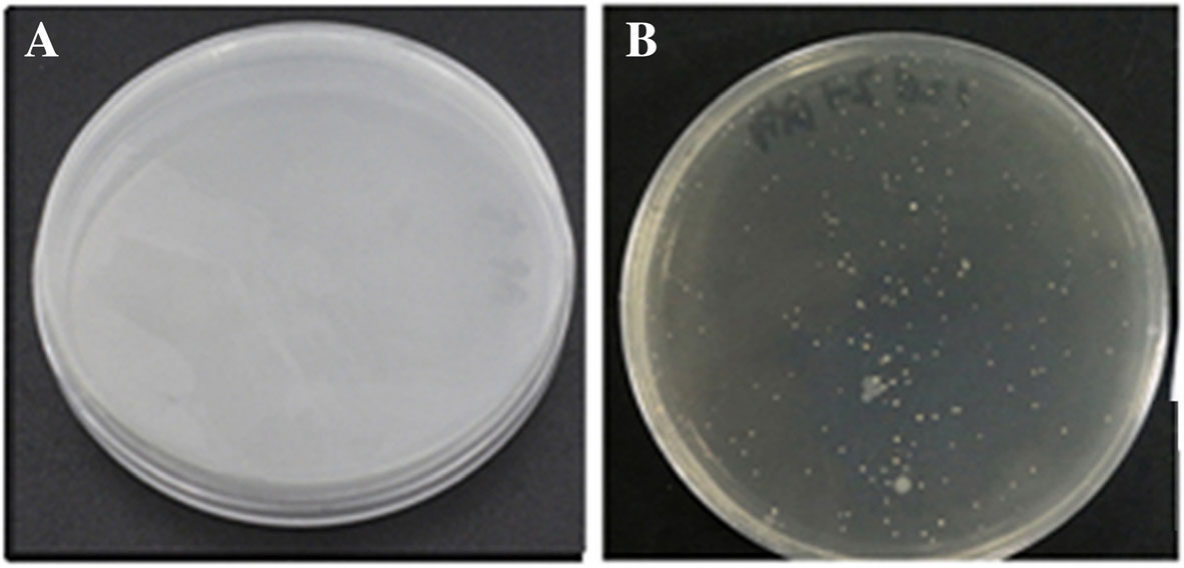
Isolation of bacteria from visceral organs. **a** Isolation of bacteria from Control group. **b** Isolation of bacteria post H9N2 AIV infection in mice

To evaluate whether intestinal injury is a common feature for chickens [7] and mice infected with H9N2 AIV strain, we firstly detected live virus in cloacal swabs. H9N2 AIV strain was detected in all mice and the peak of virus shedding was observed at 5 dpi. Histopathological analysis showed that degeneration, dissolution and necrosis of the mucosal epithelial cells, loose of the connective tissue and partial glandular atrophy were found in infected-mice at 5 dpi (Fig. 1a) and 12 dpi (Fig. 1b). While no changes were observed in control group as expected (Fig. 1c and d). Compared to the controls, the villus length was significantly reduced (*p* < 0.01, Fig. 1e) at 12 dpi and the crypt depth was significantly increased (*p* < 0.01, Fig. 1f) in infection group at 5 dpi and 12 dpi. The mucosal regular V/C was significantly reduced (*p* < 0.01, Fig. 1g) at 5 dpi and 12 dpi, suggesting that intestinal mucosa suffered varying degrees of injury after infection. Then some bacteria were isolated from liver and lung tissues of the mice at 5 and 12 dpi (Fig. 2, Table 2). Sequencing data suggested that the majority of the isolated bacteria were *Staphylococcus*, followed by a small amount of *E. coli*.

**Table 2:** Isolation of bacteria from visceral organs post H9N2 AIV infection in mice

Days of age	Tissue	Control group	Infection group
5 d	Liver	-(4/4)	+(4/4)
5 d	Lung	-(4/4)	+(4/4)
12 d	Liver	-(4/4)	+(4/4)
12 d	Lung	-(4/4)	+(4/4)

**Fig. 3:**
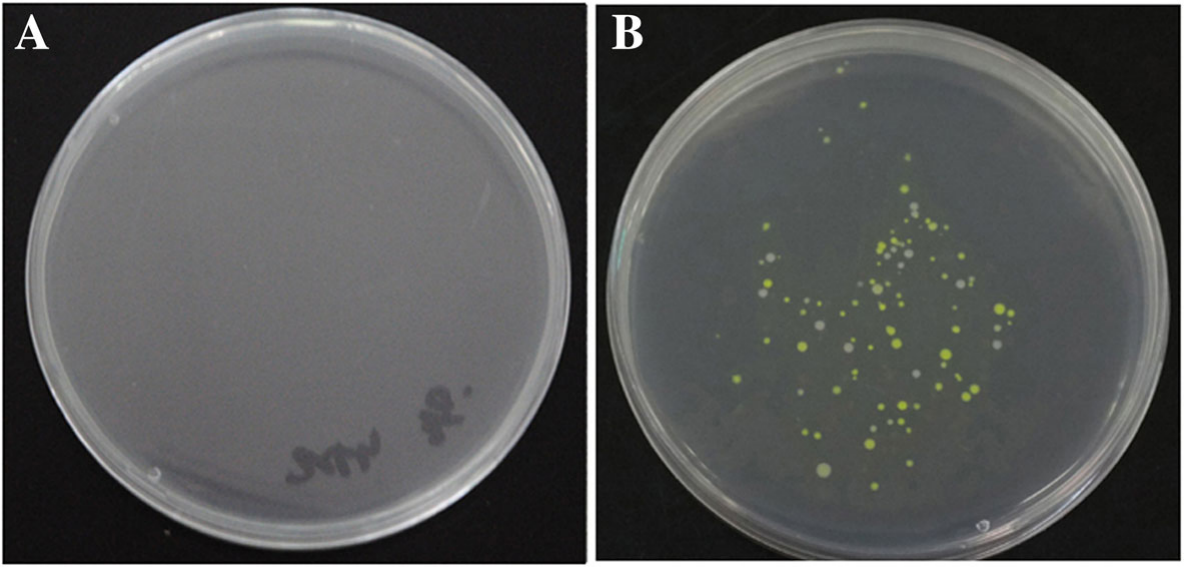
Isolation of labeled bacteria Neongreen from visceral organs. **a** Identification of labeled bacteria Neongreen from Ageratum-liquid group. **b** Identification of labeled bacteria Neongreen from Infection-Ageratum-liquid group

To test whether bacteria could transfer from the intestine through the intestinal epithelial tissue to body tissues, bacterial was isolated from the intestine cavity, liver, lung and mesentery of mice at 5 and 12 dpi by intragastrical administration of the Neongreen-labeled bacteria. The result showed that bacteria were isolated from the intestine of all mice post intragastrical administration of the Neongreen-labeled bacteria. A large number of labeled bacteria were isolated in mesentery at 36 h post intragastrical administration of the Neongreen-labeled bacteria, but none was isolated from the control group (Fig. 3, Table 3, Additional file 1, and Additional file 2). These results indicated that the bacteria transferred from the intestine through the intestinal epithelial tissue to the mesenteric tissue sites after the intragastrical administration of the Neongreen-labeled bacteria in mice. The labeled bacteria were also isolated from lungs of the infected mouse group at 24 h post intragastrical administration, while the labeled bacteria were isolated from mesentery and liver 36 and 48 h, respectively. Again, these data showed that H9N2 AIV infection might escalat bacteria translocation from intestine to the lung, mesentery and liver.

**Table 3:** Identification of Neongreen-tagged bacteria in different tissuesofNeongreen 9 groupand Infection-Neongreen groupmiceafter intragastrical administration of10 labeled bacteria

Tissue	Neongreen group				Infection**-**Neongreen group			
	12 h	24 h	36 h	48 h	12 h	24 h	36 h	48 h
intestine cavity	**+(3/3)**	**+(3/3)**	**+(3/3)**	**+(3/3)**	**+(3/3)**	**+(3/3)**	**+(3/3)**	**+(3/3)**
Lung	-(3/3)	-(3/3)	**+(3/3)**	**+(3/3)**	-(3/3)	**+(3/3)**	**+(3/3)**	**+(3/3)**
mesentery	-(3/3)	-(3/3)	-(3/3)	-(3/3)	-(3/3)	-(3/3)	**+(3/3)**	**+(3/3)**
Liver	-(3/3)	-(3/3)	-(3/3)	-(3/3)	-(3/3)	-(3/3)	-(3/3)	+**(1/3)**

### AL effectively prevented intestinal bacteria translocation post H9N2 AIV infection

Little to no bacteria was isolated from liver and lung of mice from the AL treatment group post H9N2 AIV infection. There was only one liver from one of mice of the Infection-Ageratum-liquid group at 12 dpi, which was positive with a few observable bacterial colonies on a plate (Table 4). These data suggested that AL could effectively prevent bacterial translocation to liver and lung post H9N2 AIV infection in mice. Moreover, virus titer showed that AL treatment effectively restrain H9N2 AIV invasion in ileum and lung (Additional file 3). Simultaneously, Neongreen-labeled bacterial isolation data clearly showed the Neongreen-labeled bacteria were only isolated from the intestines but others tissue of all mice subjected to either administration of Neongreen-labeled bacteria or H9N2 AIV infection followed by administration of Neongreen-labeled bacteria when feeding AL at 2, 3, 4 dpi (Table 5, Additional file 4). No Neongreen-labeled bacteria were isolated from liver, lung, and mesentery tissues of the mice from both of the treatment groups (Table 5), indicating that AL treatment effectively prevented the Neongreen-labeled bacteria translocation that otherwise would be induced post H9N2 AIV infection in mice.

**Table 4:** Isolation of bacteria from visceral organs of mice in Ageratum-liquid group and Infection-Ageratum-liquid group post H9N2 AIV infection

Days of age	Tissue	Ageratum-liquid group	Infection-Ageratum-liquid group
5 d	Liver	-(4/4)	-(4/4)
5 d	Lung	-(4/4)	-(4/4)
12 d	Liver	-(4/4)	**+**(**1/4**)
12 d	Lung	-(4/4)	-(4/4)

**Table 5:** Identification of Neongreen-tagged bacteria in different tissues of Ageratum-liquid-Neongreen group and Infection-Ageratum-liquid-Neongreen Group mice after intragastrical administration of labeled bacteria

Tissue	Ageratum-liquid-Neongreengroup				Infection-Ageratum-liquid **-**Neongreen Group			
	12 h	24 h	36 h	48 h	12 h	24 h	36 h	48 h
Intestine Cavity	**+(3/3)**	**+(3/3)**	**+(3/3)**	**+(3/3)**	**+(3/3)**	**+(3/3)**	**+(3/3)**	**+(3/3)**
Lung	-(3/3)	-(3/3)	-(3/3)	-(3/3)	-(3/3)	-(3/3)	-(3/3)	-(3/3)
Mesentery	-(3/3)	-(3/3)	-(3/3)	-(3/3)	-(3/3)	-(3/3)	-(3/3)	-(3/3)
Liver	-(3/3)	-(3/3)	-(3/3)	-(3/3)	-(3/3)	-(3/3)	-(3/3)	-(3/3)

### AL minimized intestinal mucosal injuries caused by H9N2 AIV

**Fig. 4:**
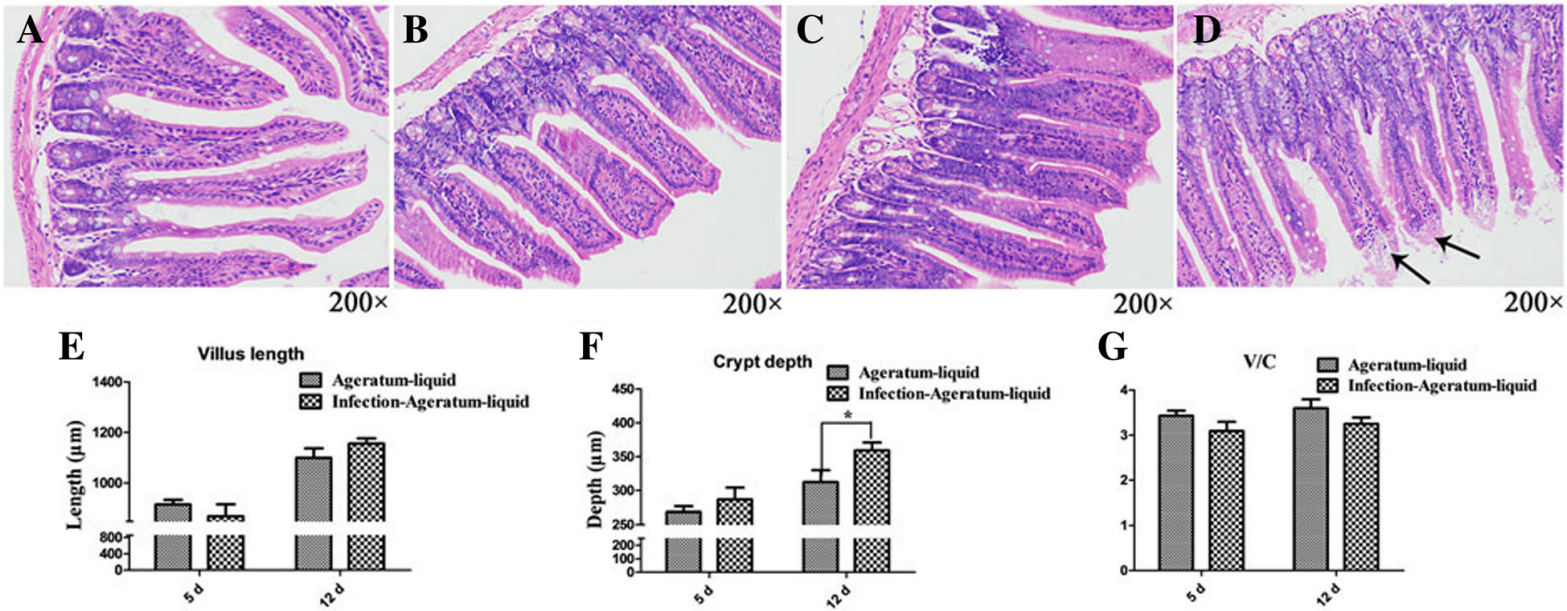
Histopathological changes in the ileal mucosa. **a**, **b** Histological features in the Ageratum-liquid groups are shown with hematoxylin and eosin staining at 5 dpi and 12 dpi; (**c**, **d**) Histological features in the Ageratum-liquid-Infection groups are shown with hematoxylin and eosin staining at 5 dpi and 12 dpi. Degeneration, dissolution and necrosis of the mucosal epithelial cells are indicated with the black arrow. **e** Measure of villus length by Image pro-plus, version 6.0 (Ageratum-liquid = 5, Infection-Ageratum-liquid = 5). **f** Measure of crypt depth by Image pro-plus, version 6.0 (Ageratum-liquid = 5, Infection-Ageratum-liquid = 5). **g** Spatial distribution of villus-length/crypt-depth of Ageratum-liquid and Infection-Ageratum-liquid groups. * *p* < 0.05

The histopathological sections of ileum of the Ageratum-liquid groups indicated that the intestinal appeared totally normal (Fig. 4a and b), and the intestinal of the Infection-Ageratum-liquid group appeared basically normal at the 5 days and 12 days post intragastrical administration (Fig. 4c and d). There was a small amount of villus epithelial cells, however, with observable necrotic or lytic in Infection-Ageratum-liquid group 12 days intragastrical administration. Simultaneously, the crypt depth of Infection-Ageratum-liquid group still was significantly increased 12 days post intragastrical administration (*p* < 0.05, Fig. 4f). While the villus length of Ageratum-liquid groups had no significant difference (Fig. 4e and f), and the V/C ultimately also was not significantly change (Fig. 1g) at 5 dpi and 12 dpi compared with Infection- Ageratum-liquid. These data showed AL could effectively relieve intestinal villus necrosis or edema and minimize intestinal mucosal damages that otherwise could be induced by H9N2 AIV infection.

### AL inhibited intestinal microflora disorder post H9N2 AIV infection

**Fig. 5:**
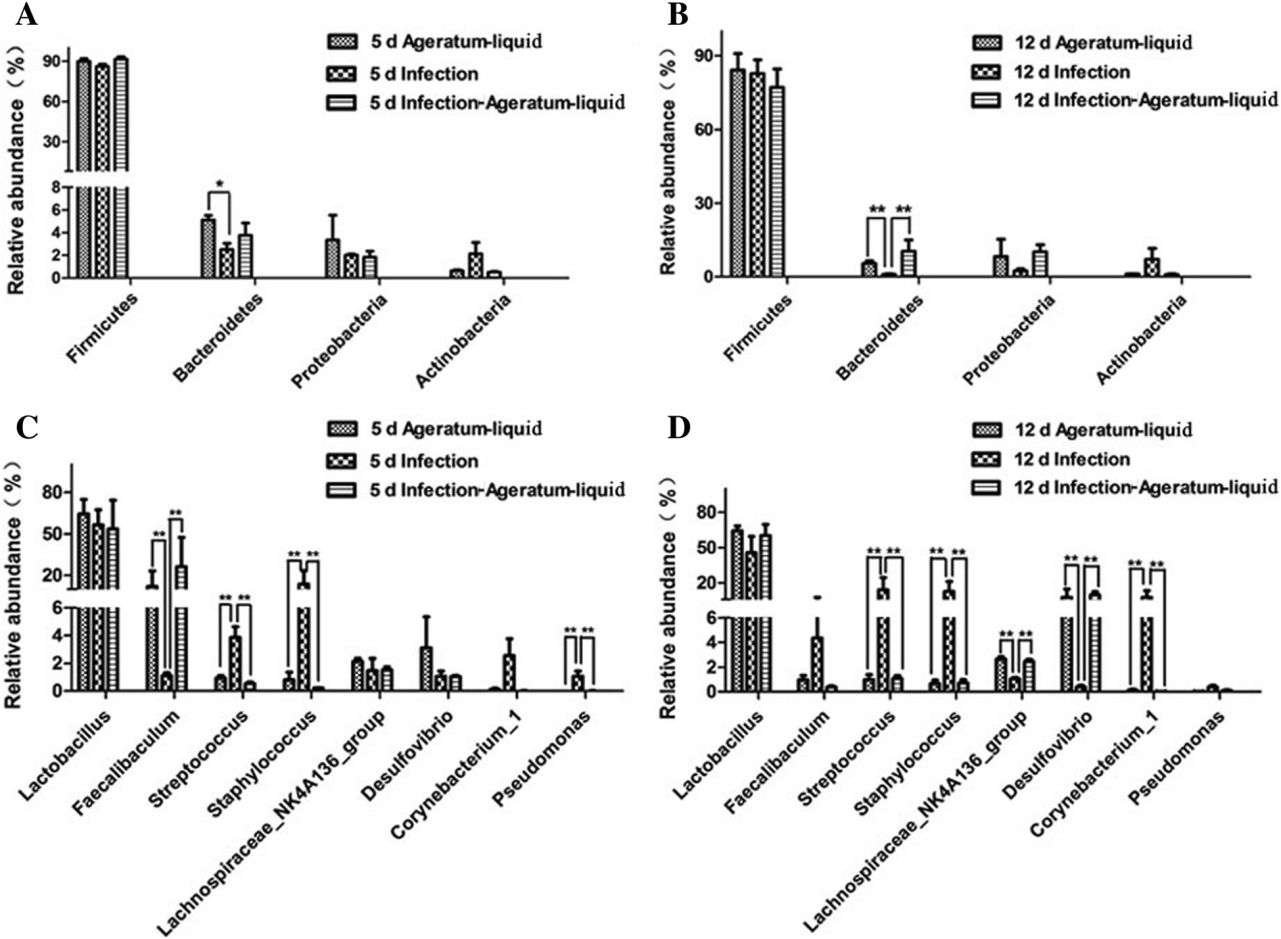
AL suppresses H9N2 AIV infection-induced intestinal flora changes. Analysis of the ileal microbiota in Ageratum-liquid, Infection and Infection-Ageratum-liquid groups by HiSeq sequencing. **a** The changes of abundance of ileal microflora at 5 dpi infection at the phylum level; **b** The changes of abundance of ileal microflora at 12 dpi at the phylum level; **c** The changes of abundance of ileal microflora at 5 dpi at the genus level; **d** The changes of abundance of ileal microflora at 12 dpi at the genus level. *: *p* <0.05, **: *p* <0.01

Intestinal microflora change was a feature of chickens or mice infected with the H9N2 AIV. 16S rRNA gene sequencing showed that the main ileal microflora was consisted of *Firmicutes*, *Bacteroidetes*, *Proteobacteria*, *Actinobacteria* in mice (Additional file 5). Principal component analysis (PCA) showed that the microbial communities in H9N2-infected and AL- treated mice could be separated using the OTU composition dataset (Additional file 6), indicating that these sediments had significantly different bacterial compositions. Compared to infection group, the abundance of *Bacteroidetes* in Ageratum-liquid group was significantly increased at 5 dpi but there was no difference in Infection-Ageratum-liquid group (Fig. 5a, *p* < 0.05). And the abundance of *Bacteroidetes* in Ageratum-liquid group and Infection- Ageratum-liquid group both were significantly increased at 12 days contrast to infection group (Fig. 5b, *p* < 0.01). Specifically, the proportion of *Staphylococcus*, *Staphylococcus* and *Pseudomonas* in Ageratum-liquid group and Infection-Ageratum-liquid group were significantly decreased at 5 days, whereas *Faecallbaculum* was significantly increased at 12 days contrast to infection group (Fig. 5c, *p* < 0.01). Moreover, compared with the infection group, the abundance of *Streptococcus*, *Staphylococcus* and *corynebacterium-1* were all significantly decreased (*p <* 0.01) at 12 dpi, while the *Lachnospiraceae_NK4A136_group* and *Desulfovibrio* were significantly up-regulated (Fig. 5d, *p* < 0.01).

These results showed that the main bacterial genus did not go through significant change in response to H9N2 AIV challenge while AL was also given at 5 and 12 dpi. This study provides evidence that AL therapeutically prevented intestinal microflora disorder in mice after H9N2 AIV infection.

## Discussion

H9N2 AIV, a low-virulent virus, is widely found in some avian species, and mammals, causing significant losses due to diarrhea and secondary infections [1, 2, 15]. There are many studies reporting the effects of H9N2 AIV on the respiratory tract [16, 17], but few reports focus on the effect of H9N2 AVI on the gastrointestinal tract. Stable intestinal morphology and physiological function are the basis of the intestinal health. It has been reported that short villus height and deep crypt depth means the poor intestinal function. The higher to the V/C value, the healthier to the intestine [18, 19]. In this study, the villus of ileum became shorter, the crypt became deeper, and V/C was down-regulated significantly after 5 and 12 dpi, indicating that mice infected with H9N2 AIV damaged the structure of villus and affected the function of nutritional absorption. The change of villi epithelial cells could be directly observed through pathological sections, showing, in accordance with previous observations [7, 20], H9N2 AIV infection could destroy the intestinal barrier structure, weaken digestion and absorption capacity, and further may affect the body’s immune capacity, resulting in that conditional pathogens might reach to pathogenic conditions.

It has been reported that H9N2 AIV infection could change the structure of intestinal flora with beneficial bacteria reduction and damage the structure of intestinal barrier in mice or chicken [7, 20, 21]. At 5 dpi, broiler chickens were greatly reduced in beneficial bacteria such as *Lactobacillus*, and the number of opportunistic pathogens increased, especially the conditional pathogens *Escherichia* abundance exceeded 40%. Similarly, H9N2 AIV infected mice also showed some changes in bacterial population, resulting in increased abundance of *Proteobacteria* and *Actinomycetes*. Especially, beneficial *Lactobacillus* was significantly down-regulated, and *Streptococcus*, *Staphylococcus* and Coryneba*cterium-1* were significantly up-regulated at 12 dpi. As we know, the etiological relationship between gastrointestinal diseases and intestinal microflora was relatively constant [22, 23]. We proposed that the structure disruption of mouse intestinal flora caused by H9N2 AIV probably took shape a bacterial environment to trend to critical pathological conditions. Meanwhile, we isolated plenty of *Staphylococcus* and a few *E. coli* from liver and lung in infected mice. Those findings provide clues that these microbes might be from translocation of intestine. The isolation of Neongreen-labeled bacteria confirmed this hypothesis. The labeled bacteria transferred from the intestine through the intestinal epithelial tissue to the mesenteric tissue sites in 36 h and lungs in 24 h post intragastrical administration of Neongreen-labeled bacteria. These results indicated that H9N2 AIV promotes some bacterium translocation into body, then invaded other tissues through body fluid circulation and so on, leading to bacterial infection. Which bacteria can synergize with H9N2 AIV co-infection and how does this synergy work? These require further study. In addition, the labeled bacteria were also isolated from lungs of the infected mouse group 24 h post intragastrical administration, indicating specific intestinal bacteria may also be able to infect damaged lungs by the digestive tract.

Patchouli is an edible plant, which broadly grows in China and is the main ingredient of AL. As one of the 50 fundamental herbs, Patchouli is used in popular medicine to viral, fungal, and bacterial infections [24–26]. Magnolia officinalis (Chinese name: Houpo) was an important constituent of AL and rich in Honokiol or magnolol, isomers of neolignans [27, 28], which was reported that honokiol have function of anti-oxidative, anti-inflammatory, anti-tumor and anti-microbial properties [29–34]. A previous review showed that the mechanisms of kampo medicines including some components of AL were involved in regulating the intestinal motility by NO or 5-HT3 receptor pathways and anti-inflammatory [35]. As a thousand-year-old formula, AL combines each component biological and pharmacological property and is an efficiency and low toxicity natural medicines. It is reported that the AL can increase the number of Lactobacillus, Bifidobacterial in mammal gut, reduce intestinal permeability in acetic acid-induced PI-IBS, regulate CD4+ and CD8+ cells in Peyer’s patch and suppress TNF-alpha levels in enteric homogenates to improve the diarrhea caused by Salmonella typhimurium in mice [9, 10, 36, 37, 38]. Our experiment found AL can effectively minimize the H9N2 AIV infection-induced intestinal mucosal injuries and alleviate bacterial flora disorder, suggesting that AL no only might somehow improve the immunity and anti-H9N2 AIV capabilities of host, but also repair intestinal barrier to prevent secondary infections. Thus, as a low resistance and toxicity natural medicine, traditional Chinese medicine has many pharmacological activities and could be applied to treat many diseases as potential antibiotic substitutes. Those need to further research.

Meanwhile, little to no bacteria was isolated from liver and lung of mice after H9N2 AIV infection in Ageratum-liquid group and Infection-Ageratum-liquid group. Moreover, no Neongreen-labeled bacteria were isolated from liver, lung, and mesentery tissues of the mice from both of the treatment groups, but the Neongreen-labeled bacteria were isolated from the intestines of all mice subjected to either administration of Neongreen-labeled bacteria or H9N2 AIV infection followed by administration of Neongreen-labeled bacteria, indicating that gut bacterial translocation to the baby might play important roles during H9N2 AIV infection process and AL treatment effectively prevented the bacteria translocation that otherwise would be induced post H9N2 AIV infection in mice.

## Conclusions

As a low-virulent virus, AIV H9N2 causes mild respiratory illness, but usually involved secondary bacterial infection in mammals and chicken [1, 3, 5]. Our study explicitly demonstrated that H9N2 AIV infection could damage the ileal mucosal barrier and induce the disturbance of the intestinal flora in BALB/c mice resulting in translocation of intestinal bacteria. Our data also illustrated that the traditional Chinese medicine AL can effectively prevent bacterial translocation following H9N2 infection. These findings are of important theoretical and practical significance in minimizing H9N2 AIV clinical damages, including secondary infections.

## Additional files

Additional file 1: *E.coli* (Neongreen-tagged bacteria) CPU in the tissue of 1 Neongreen group miceafter intragastrical administrationof labeled bacteria. (PDF 12 kb)

Additional file 2: *E.coli* (Neongreen-tagged bacteria) CPU in the tissue of 1 Infection**-**Neongreen groupmiceafter intragastrical administrationof labeled bacteria. (PDF 12 kb)

Additional file 3: Intestine cavity H9N2 virus titer in mice infected with 300 μL of 10^6.1^ EID_50_/0.1 mL H9N2 AIV. Virus titer of ileum (A) and lung (B) were determined at 5 dpi and 12 dpi using TCID_50_ in MDCK cells. (JPG 41 kb)

Additional file 4: *E.coli* (Neongreen-tagged bacteria) CPU in the tissue of Ageratum-liquid-Neongreengroupand 1 Infection-Ageratum-liquid **-**Neongreen Groupmiceafter intragastrical administrationof labeled bacteria. (PDF 17 kb)

Additional file 5: The ileal microbiota was analyzed by sequencing using the Illumina HiSeq system. The relative abundance of the bacterial phylum is displayed. (JPG 318 kb)

Additional file 6: AL inhibited H9N2 AIV infection-induced intestinal microflora disorder. Principal component analysis (PCA) of OTU composition data is displayed using software R (v3.1.1). (JPG 121 kb)

## Abbreviations

AL: Ageratum-liquid
dpi: Day post infection
h: Hour
H7N9: Avian influenza A (H7N9) virus
H9N2 AIV: Avian influenza virus subtype H9N2
V/C: Villus-length/crypt-depth
