# Supplementary material for: Effect of feeding Chinese herb medicine ageratum-liquid on intestinal bacterial translocations induced by H9N2 AIV in mice
Source: Virol J. 2019 Feb 21;16:24. doi: 10.1186/s12985-019-1131-y (PMC6385471; doi:10.1186/s12985-019-1131-y)
Supplement: Supplementary file 1 — E.coli (Neongreen-tagged bacteria) CPU in the tissue of 1 Neongreen group miceafter intragastrical administrationof labeled bacteria. (PDF 12 kb) [file 12985_2019_1131_MOESM1_ESM.pdf]

Supplementary Material 1. *E.coli* (Neongreen-tagged bacteria) CPU in the tissue of Neongreen group mice after intragastrical administration of labeled bacteria

Tissue	Neongreen group			
	12 h	24 h	36 h	48 h
intestine cavity	1008.3±240.2	691.7±364.3	816.7±289.8	258.3±137.7
Lung	0	0	83.3±52	100±90.1
mesentery	0	0	0	0
Liver	0	0	0	0
