# Supplementary material for: Effect of feeding Chinese herb medicine ageratum-liquid on intestinal bacterial translocations induced by H9N2 AIV in mice
Source: Virol J. 2019 Feb 21;16:24. doi: 10.1186/s12985-019-1131-y (PMC6385471; doi:10.1186/s12985-019-1131-y)
Supplement: Supplementary file 2 — E.coli (Neongreen-tagged bacteria) CPU in the tissue of 1 Infection-Neongreen groupmiceafter intragastrical administrationof labeled bacteria. (PDF 12 kb) [file 12985_2019_1131_MOESM2_ESM.pdf]

Supplementary Material 2. *E.coli* (Neongreen-tagged bacteria) CPU in the tissue of Infection-Neongreen group mice after intragastrical administration of labeled bacteria

Tissue	Infection-Neongreen group			
	12 h	24 h	36 h	48 h
intestine cavity	996.6±287.6	1133.3±362.6	1191.7±563.4	8658.3±5405.7
Lung	0	508.3±267.3	2475±1412.7	4916.7±2350.7
mesentery	0	0	833.3±274.2	616.7±314.6
Liver	0	0	0	41.7±72.2
