# Supplementary figures and images for: Effect of feeding Chinese herb medicine ageratum-liquid on intestinal bacterial translocations induced by H9N2 AIV in mice
Source: Virol J. 2019 Feb 21;16:24. doi: 10.1186/s12985-019-1131-y (PMC6385471; doi:10.1186/s12985-019-1131-y)

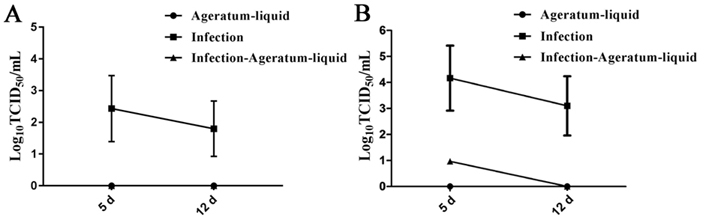

Supplement: Supplementary file 3 — Intestine cavity H9N2 virus titer in mice infected with 300 μL of 106.1 EID50/0.1 mL H9N2 AIV. Virus titer of ileum (A) and lung (B) were determined at 5 dpi and 12 dpi using TCID50 in MDCK cells. (JPG 41 kb) [file 12985_2019_1131_MOESM3_ESM.jpg]

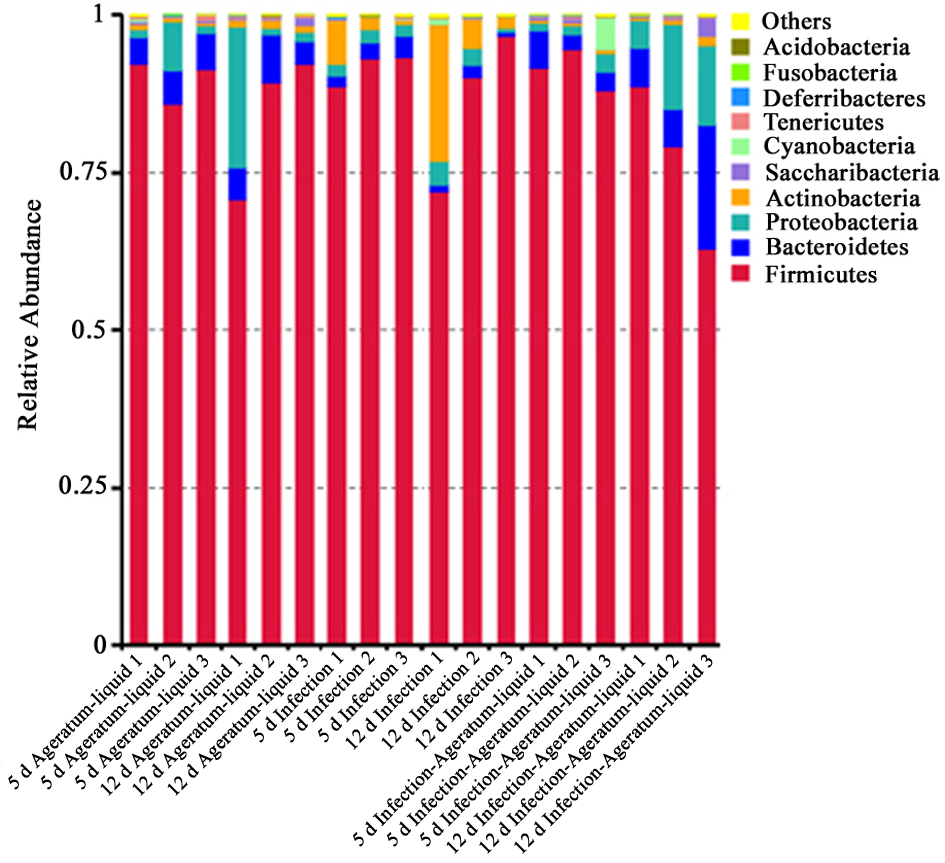

Supplement: Supplementary file 5 — The ileal microbiota was analyzed by sequencing using the Illumina HiSeq system. The relative abundance of the bacterial phylum is displayed. (JPG 318 kb) [file 12985_2019_1131_MOESM5_ESM.jpg]

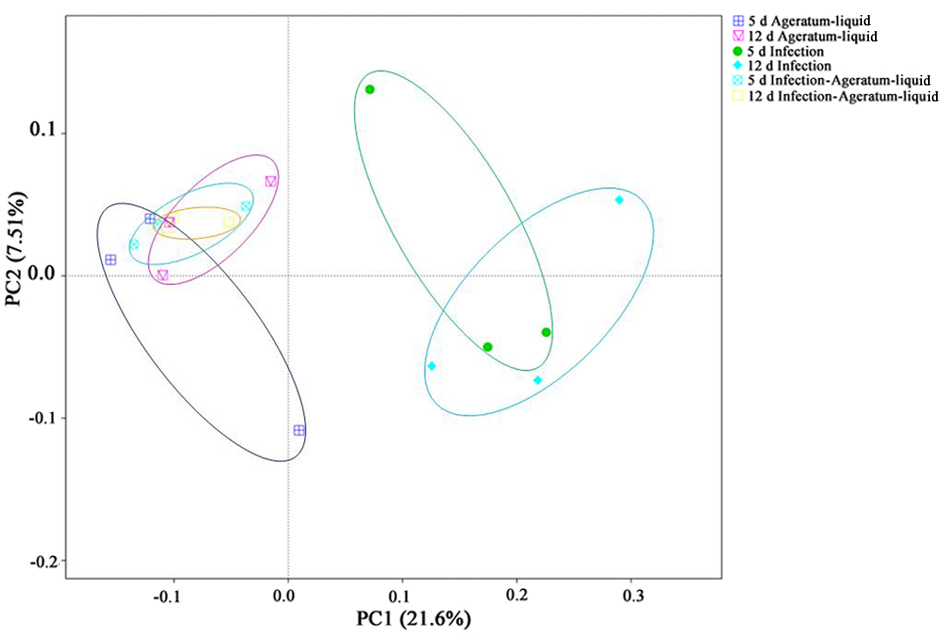

Supplement: Supplementary file 6 — AL inhibited H9N2 AIV infection-induced intestinal microflora disorder. Principal component analysis (PCA) of OTU composition data is displayed using software R (v3.1.1). (JPG 121 kb) [file 12985_2019_1131_MOESM6_ESM.jpg]
