# Supplementary material for: Effect of feeding Chinese herb medicine ageratum-liquid on intestinal bacterial translocations induced by H9N2 AIV in mice
Source: Virol J. 2019 Feb 21;16:24. doi: 10.1186/s12985-019-1131-y (PMC6385471; doi:10.1186/s12985-019-1131-y)
Supplement: Supplementary file 4 — E.coli (Neongreen-tagged bacteria) CPU in the tissue of Ageratum-liquid-Neongreengroupand 1 Infection-Ageratum-liquid -Neongreen Groupmiceafter intragastrical administrationof labeled bacteria. (PDF 17 kb) [file 12985_2019_1131_MOESM4_ESM.pdf]

Supplementary Material 4. *E.coli* (Neongreen-tagged bacteria) CPU in the tissue of Ageratum-liquid-Neongreen group and Infection-Ageratum-liquid -Neongreen Group mice after intragastrical administration of labeled bacteria

Tissue	Ageratum-liquid-Neongreen group				Infection-Ageratum-liquid -Neongreen Group			
	12h	24h	36h	48h	12h	24h	36h	48h
Intestine Cavity	1125±319.2	450±477	108.3±52	66.7±52	791.7±623.7	800±354.4	200±156.1	91.7±63
Lung	0	0	0	0	0	0	0	0
Mesentery	0	0	0	0	0	0	0	0
Liver	0	0	0	0	0	0	0	0
